# Molecular Mechanisms of Hepatocarcinogenesis Following Sustained Virological Response in Patients with Chronic Hepatitis C Virus Infection

**DOI:** 10.3390/v10100531

**Published:** 2018-09-28

**Authors:** C. Nelson Hayes, Peiyi Zhang, Yizhou Zhang, Kazuaki Chayama

**Affiliations:** 1 Department of Gastroenterology and Metabolism, Graduate School of Biomedical & Health Sciences, Applied Life Sciences, Institute of Biomedical & Health Sciences, Hiroshima University, 1-2-3 Kasumi, Minami-ku, Hiroshima 734-8551, Japan; g170261@hiroshima-u.ac.jp (P.Z.); chayama@hiroshima-u.ac.jp (K.C.); 2 Research Center for Hepatology and Gastroenterology, Hiroshima University, Hiroshima 734-8551, Japan; 3 Department of Neurosurgery, Icahn School of Medicine at Mount Sinai, New York, NY 10029, USA; zhangyizhou10@yahoo.co.jp

**Keywords:** hepatocellular carcinoma, hepatitis C virus, sustained virological response, direct-acting antiviral agents, interferon, recurrence, fibrogenesis, reactive oxygen species

## Abstract

Despite the success of direct-acting antiviral (DAA) agents in treating chronic hepatitis C virus (HCV) infection, the number of cases of HCV-related hepatocellular carcinoma (HCC) is expected to increase over the next five years. HCC develops over the span of decades and is closely associated with fibrosis stage. HCV both directly and indirectly establishes a pro-inflammatory environment favorable for viral replication. Repeated cycles of cell death and regeneration lead to genomic instability and loss of cell cycle control. DAA therapy offers >90% sustained virological response (SVR) rates with fewer side effects and restrictions than interferon. While elimination of HCV helps to restore liver function and reverse mild fibrosis, post-SVR patients remain at elevated risk of HCC. A series of studies reporting higher than expected rates of HCC development among DAA-treated patients ignited debate over whether use of DAAs elevates HCC risk compared to interferon. However, recent prospective and retrospective studies based on larger patient cohorts have found no significant difference in risk between DAA and interferon therapy once other factors are taken into account. Although many mechanisms and pathways involved in hepatocarcinogenesis have been elucidated, our understanding of drivers specific to post-SVR hepatocarcinogenesis is still limited, and lack of suitable in vivo and in vitro experimental systems has hampered efforts to examine etiology-specific mechanisms that might serve to answer this question more thoroughly. Further research is needed to identify risk factors and biomarkers for post-SVR HCC and to develop targeted therapies based on more complete understanding of the molecules and pathways implicated in hepatocarcinogenesis.

## 1. Introduction

Hepatocellular carcinoma (HCC) is the most common type of primary liver cancer as well as the fastest growing cause of cancer-related mortality worldwide [1]. Incidence of HCC is 2–4 times more common in men than in women and is the 5th and 7th most common cause of cancer-related death in men and women, respectively. Up to 850,000 new cases are diagnosed each year, and this number is expected to grow, especially in hepatitis B virus (HBV)-endemic regions, including sub-Saharan Africa and Southeast Asia that together account for 85% of HCC cases. Overall, HCC incidence and mortality in developing countries is double that of developed countries. While HBV vaccination has reduced the number of new infections, there remains no curative treatment for chronic HBV, greatly increasing the risk of HCC in these patients. A number of other etiologies are known or suspected to cause HCC, including hemochromatosis, obesity, excessive alcohol consumption, inflammation, primary biliary cirrhosis, α-1 antitrypsin deficiency, Wilson’s disease, and exposure to carcinogens such as aflatoxin B_1_, thorotrast, polyvinyl chloride, and carbon chloride. However, hepatitis C virus (HCV) infection is the leading cause of HCC in developed countries and is responsible for the recent overall rise in HCC cases [2,3,4]. Chronic infection with HCV is the major risk factor for HCC in Europe and North America and is responsible for about 25% of HCC cases. Unlike HBV, which establishes itself in a durable form in the nuclei of hepatocytes and is capable of triggering genomic instability by integrating into host genomic DNA, HCV is an RNA virus that resides only in the cytoplasm and must replicate continuously to maintain infection [5]. While there remains no preventative vaccine, HCV is now widely considered a curable disease, with rates above 90% for sustained virological response (SVR), defined as undetectable HCV viremia 24 weeks after the end of therapy. Widespread adoption of direct-acting antiviral (DAA) therapy promises to reduce the incidence of HCC in HCV-infected patients. While the number of new cases of HCV has been decreasing since the introduction of improved blood screening measures in the 1980s, HCC infection may not develop for 20–40 years after infection [6]. In fact, the peak of HCV-related HCC may be imminent, with more than a million “baby boomers” projected to develop cirrhosis, decompensated liver disease, or HCC by 2020 at a cost of $8.6 billion in the United States alone [7,8]. Patients with chronic HCV should be treated as early as possible to reduce the risk of HCC, but even successful clearance of the virus may not prevent progression to HCC, and HCC may develop in some patients up to 10 years after SVR.

### 1.1. Detection and Treatment of HCC

Several treatments are available for HCC, but early detection remains a critical predictor of survival. Liver tumors are usually multicentric and may occur as one or more discrete lesions or as diffuse growth. HCC is uncommon before age 40 and incidence peaks around age 70. Without treatment, HCC proliferates aggressively and results in liver failure and death, with mean survival time between 6 to 20 months. While in the past, HCC was often detected at an advanced stage accompanied by abdominal pain, weight loss, and decompensated liver disease, screening of cirrhotic patients with respect to alpha-fetoprotein (AFP) levels and cross-sectional imaging with ultrasonography, computed tomography, magnetic resonance imaging, and angiography has resulted in earlier detection and greater treatment options. Resection may be effective when detected early, but many patients are ineligible due to cancer stage or degree of liver function, and the benefit is often temporary, with a 70% rate of HCC recurrence five years after resection. Similarly, HCC is one of the most common indications for liver transplantation, which, while potentially curative, is constrained by organ availability and access. Advances in ablative therapies such as radiofrequency ablation, percutaneous ethanol injection, transcatheter arterial chemoembolization (TACE), and hepatic arterial infusion chemotherapy (HAIC) has led to improved prognosis in HCC patients, but survival rates remain poor for patients with complications such as portal vein thrombosis and refractoriness to TACE [9,10,11,12]. Treatment with the multikinase inhibitor sorafenib has been shown to extend overall survival time by several months in patients with advanced HCC, but its effect is regarded as insufficient [9]. The immune checkpoint inhibitor nivolumab shows promise in prolonging survival in patients who fail to respond to sorafenib by modulating the immune response [13]. For advanced HCC patients with portal vein thrombosis, yttrium-90 radioembolization has been shown to be safe and effective with survival times of up to 13.3 months for patients with Child-Pugh class A and 3.9 months for Child-Pugh class B [14,15].

### 1.2. Chronic Hepatitis C Virus Infection

Based on country-level disease burden models, there are estimated to be 71.1 million chronic HCV carriers worldwide, representing 1% of the world’s population, lower than previous World Health Organization estimates but nonetheless posing an enormous worldwide public health challenge [16,17]. There is no HCV vaccine, and only a fraction of patients achieve spontaneous clearance of the virus following acute infection with HCV, and without treatment most patients progress to chronic HCV infection and endure lifelong infection [18]. Although the number of new HCV infections has fallen greatly since the introduction of changes in blood screening implemented in 1990, there are estimated to be 3–4 million new cases per year, and some subgroups, such as injection drug users, remain at high risk of new infection as well as reinfection [19].

HCV is an enveloped, positive-sense, single-stranded RNA virus in the Flaviviridae. The 9.6 kb RNA genome encodes a single 3011 amino acid polyprotein that is cleaved by host and viral proteases into three structural proteins (Core, E1, and E2) and 7 non-structural proteins (NS1, NS2, NS3, NS4A, NS4B, NS5A, and NS5B) [20]. As a hepatotropic virus with high species and tissue specificity, HCV enters and replicates specifically within human hepatocytes. HCV is highly variable with at least 6 genotypes varying by 30–35% and numerous multiple sub-genotypes that vary by 20–25%. HCV genotypes and sub-genotypes are known to respond differently to treatment and may possess different barriers to resistance as well as pose different degrees of HCC risk. Within an individual, numerous quasispecies are thought to coexist at different frequencies, and quasispecies composition may vary even within HCC tumors and adjacent tissue.

Although HCV is considered non-cytopathic, HCV infection inflicts immune-mediated hepatic injury leading to chronic hepatitis, cirrhosis, and hepatocarcinogenesis. Inflammation resulting from HCV infection leads to fibrosis, which progresses to cirrhosis in 20–30% of patients over a span of 20 to 40 years. 1–2% of patients with cirrhosis eventually develop HCC [21]. Because HCV carriers, particularly elderly patients with advanced hepatic fibrosis, are at high risk of liver carcinogenesis, such patients need therapy to eradicate HCV as early as possible. Patients with cirrhosis who achieve SVR have been shown to have improved survival times and respond better to treatment for ascites and portal hypertension. Interferon therapy, which was later modified to use pegylated interferon in combination with ribavirin, achieved SVR rates near 50% for difficult to treat genotype 1 patients. Because of the severe side effects of interferon and ribavirin, older patients and patients with cirrhosis, impaired renal function, HIV/HBV coinfection, or other complications were often ineligible. Therefore, patients treated with interferon were more likely to be younger and healthier than untreated patients. Fortunately, the introduction of interferon-free DAAs pushed SVR rates to 90%, with many clinical trials reporting SVR rates approaching 100%.

**Figure 1:**
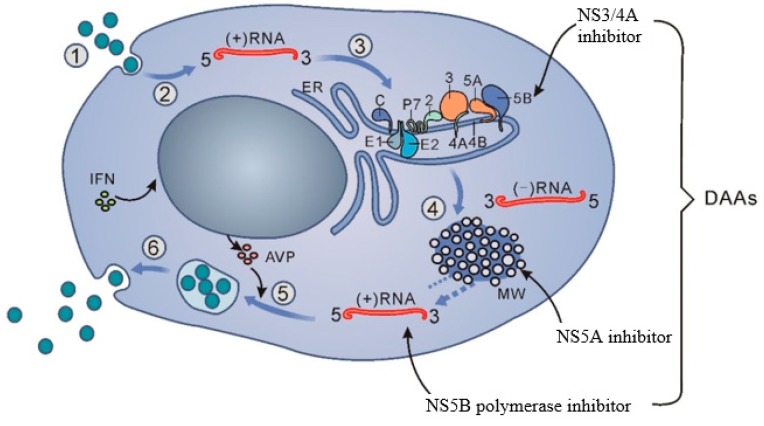
The hepatitis C virus life cycle and targets of interferon and direct-acting antiviral therapies. (1) cell entry, (2) uncoating, (3) HCV RNA translation and cleavage of the polyprotein, (4) RNA replication, (5) assembly, and (6) release.

While the NS3 protease inhibitors telaprevir and boceprevir had been approved earlier for use in combination with peg-interferon and ribavirin, the first interferon-free DAA therapy was not approved until 2014 with the introduction of daclatasvir and asunaprevir in Japan. As such, there is not enough data yet to assess the long-term effects of DAAs on HCC risk. In addition, development of effective pan-genotypic DAAs with high tolerability and a high barrier to resistance has proceeded rapidly over the past several years, presenting a changing landscape with a number of competing therapies currently approved. However, current DAA combination therapies include some combination of at least two agents representing four drug classes, NS3 protease inhibitors, NS5A inhibitors, and two types of NS5B polymerase inhibitors (Figure 1). Current DAA combination therapies include sofosbuvir plus ledipasvir (SOF/LDV), ombitasvir plus paritaprevir/ritonavir (OMV/PTV/r), elbasvir plus grazoprevir (EBV/GPV), and glecaprevir plus pibrentasvir. With some variation in dose or composition depending on the genotype or presence of specific resistance-associated variants, DAA therapy is largely pan-genotypic, and nearly all patients are eligible. However, DAAs are not a panacea; high costs, limited access throughout much of the world, and a low but potentially problematic risk of antiviral resistance pose challenges to public health. For example, costs and availability of sofosbuvir vary widely among nations, but 90% of HCV carriers live in areas with limited resources. In principle, DAA resistance can be countered using different combinations of DAAs with non-overlapping resistance profiles, but many DAAs are only available or approved as co-formulated tablets, limiting flexibility in treatment, and currently almost no DAA therapies are effective against HCV NS5A p32 deletion mutants, and the frequency of other NS5A resistance-associated variants in treatment-naive patients raises questions about the continued long-term efficacy of current NS5A inhibitors. It is hoped that development of combination therapies involving agents that target host factors required for HCV replication will reduce the risk of resistance, but the development of host-targeting agents has been slow. Furthermore, the actual number of HCV carriers is probably higher than estimated because many patients remain undiagnosed.

### 1.3. Differences between Treatment with DAA and IFN

Patients who achieve SVR with IFN or DAAs have been shown to have improved life expectancy and reduced incidence of HCC relative to untreated patients and those who fail to clear the virus [22,23,24,25,26,27]. SVR is associated with improved quality of life and lower risk of hepatic decompensation, coagulopathy, and ascites [28]. Therefore, the widespread adoption of highly effective DAA treatments is expected to reduce the risk of HCV-related HCC [29]. However, while SVR reduces the incidence of HCC relative to untreated patients, it does not eliminate the risk, and in a small percentage of patients, fibrosis levels continue to increase, leading to cirrhosis or HCC [30,31,32,33].

Several recent reports have raised concerns that the risk of post-SVR HCC is higher in patients treated with DAAs than in patients treated with interferon plus ribavirin, particularly among patients with advanced liver cirrhosis or a prior history of HCC [30,31,34]. Reig et al. reported an unexpectedly high rate of HCC recurrence (16 cases, 3 deaths) in a group of 53 post-SVR patients who had prior history of HCC but who had no evidence of HCC at the time of DAA therapy [30]. Conti et al. reported 26 cases of HCC, 17 of which were recurrences, during 24 week follow-up in a study of 344 consecutive cirrhotic patients [31]. Prior history of HCC and Child-Pugh score were independent risk factors for HCC, and the authors note that short-term risk of HCC remains high despite SVR, especially in younger patients with severe fibrosis. Kozbial et al. also observed a higher than expected frequency of HCC of up to 8% after successful DAA therapy [34].

The reason for the alarming HCC rates reported in these studies is not clear, although differences in the mechanisms of IFN-versus DAA-mediated HCV clearance have been proposed to explain any potential difference in HCC risk. IFN plus ribavirin therapy is associated with severe side effects due to its systemic effects and is typically administered over far longer periods (up to 48 or even 72 weeks) compared to the much shorter 8–24 week duration and far milder side effects of DAA therapy. One practical consequence of this difference is that patients who were treated with IFN were likely to be younger and healthier, and patients with cirrhosis or other comorbidities or complications were typically ineligible. As a result, DAA-treated patients are increasingly likely to be older and have more advanced fibrosis, resulting in a higher overall expected incidence of HCC, regardless of treatment [35]. Furthermore, SVR rates with IFN were only about 50% for HCV genotype 1, and likelihood of SVR rate was strongly associated with *IL28B* genotype, whereas successfully treated DAA patients represent a far more diverse patient population. However, interferon is a potent systemic immune modulator that helps to limit cell proliferation and might therefore be protective against malignant transformation [36,37]. It has been suggested that by eliminating HCV, DAAs might alter the risk of HCC by promoting restoration of innate immunity and down-regulation of type II and III interferons and their associated receptors and target genes, as well as reduced levels of miR-122, which might result in relaxed immune surveillance and a more permissive state for proliferation of malignant cells [34]. A protective effect of interferon against HCC has been observed in chronic HBV patients in which HCC incidence was higher among patients treated with nucleos(t)ide analogues compared to those treated with peg-interferon [38]. Eradication of HCV via interferon, unlike DAAs, also helps to restore p53 activity and the ER stress response [39,40]. Regardless of the potential mechanism, millions of patients are expected to be treated with DAA therapy, and clear evidence of a direct or indirect effect of DAA therapy on risk of HCC development or recurrence might influence treatment decisions, such as which patients should be most closely monitored and whether DAA treatment should be delayed until after transplantation to reduce risk of HCC in patients awaiting orthotopic liver transplantation [34].

Fortunately, a number of recent prospective and retrospective studies have reexamined the risks of DAA versus IFN therapy on HCC development and failed to confirm these worrisome earlier findings (Table 1 and Table 2). The general consensus now appears to be that patients who achieve SVR with DAA therapy do not have a significantly greater HCC risk than those treated with IFN. In a large multicenter retrospective study in France involving 1270 patients with cirrhosis, Nahon et al. reported HCC rates of 5.9% among patients treated with DAAs, 3.1% in patients treated with IFN, and 12.7% among patients who did not achieve SVR [41]. However, DAA-treated patients were older with higher rates of diabetes, portal hypertension, and severely impaired liver function compared to IFN-treated patients. After accounting for confounding effects of patient background using a time-dependent Cox regression model weighted by the inverse probability of treatment and censoring, the authors found no significant increase in risk due to DAA treatment. Another report examining HCC recurrence with respect to treatment type found no evidence of elevated risk following DAA therapy in patients previously treated for HCC [42]. Nagata et al. also reported similar rates of HCC after DAA or IFN therapy using a propensity score-matched analysis [43]. The authors observed that post-treatment levels of Wisteria floribunda agglutinin positive Mac-2 binding protein (WFA + M2BP) were independently associated with HCC after SVR and advocate the potential use of this protein as a biomarker. Yasui et al. also reported that WFA + M2BP levels was predictive of HCC in patients with no previous HCC history [44]. Motoyama et al. suggested that failure to observe improvement after SVR due to hepatic stellate cell activation is a risk factor for post-SVR HCC [45]. In a large retrospective cohort study of more than 10,000 patients who achieved SVR, among whom 100 developed HCC, El-Serag found that HCC risk is significantly reduced following SVR, but the authors caution that the risk remains relatively high (0.33%), particularly among patients with cirrhosis, diabetes, or genotype 3 infection and in patients older than 64 [40,46]. Results from these studies suggest that the apparent elevation in HCC risk associated with DAAs can be attributed in part due to differences in patient characteristics and screening intensity [41]. In a small study on HCC recurrence, Zavaglia et al. found only one case of HCC recurrence out of 31 patients [47]. The authors speculate that the low rate of HCC recurrence could be explained in part by the long (median 19 months) interval between HCC treatment and start of DAA therapy. Although DAAs do not appear to increase the risk of HCC, patients should be treated as early as possible to minimize fibrosis progression, and patients with advanced fibrosis or cirrhosis should be closely monitored for HCC [48].

## 2. Molecular Mechanisms of HCC

The molecular mechanisms underlying HCC development have not been fully elucidated but likely reflect the cumulative effect of various interacting host, viral, and environmental factors over a period of 20–40 years [8]. Despite the diversity of HCC etiologies, several factors in common have been linked to HCC development, including inflammation, necrosis, fibrosis, and regeneration. While HBV-related HCC may develop in patients without cirrhosis due to the activity of the HBV X protein, HCV-related HCC development is strongly correlated with fibrosis stage and primarily occurs in the context of cirrhosis [70]. Unlike most cancers, which result following the accumulation of a series of mutations affecting oncogenes and tumor suppressor genes, HCV proteins have been shown to induce carcinogenesis without accompanying genetic alternations (“non-Vogelstein-type” carcinogenesis) [71]. Despite these differences, the late stages of hepatocarcinogenesis are thought to converge on common mechanisms regardless of etiology [72]. Unless otherwise noted, many of the following mechanisms are not necessarily specific to post-SVR HCC due to the current lack of detailed understanding of drivers of post-SVR hepatocarcinogenesis.

Liver tumors are monoclonal and are thought to derive from hepatic stem cells that undergo cycles of damage and regeneration in response to HCV-mediated liver injury that favors establishment of a fibrogenic pro-oncogenic microenvironment [73]. The presence of HCV results in a state of chronic inflammation that disrupts homeostasis and induces oxidative stress, altered signaling cascades, insulin resistance, hepatosteatosis, and progressive fibrogenesis [74]. Immune-mediated inflammation triggers production of reactive oxygen species (ROS) that disrupt hepatocyte metabolism and damage genome integrity, resulting in cell death. Accumulation of free fatty acids also causes ER stress and mitochondrial damage, and oxidative stress up-regulates collagen 1 expression and promotes inflammation through lipid peroxidation [75]. Sustained cellular proliferation in the presence of inflammatory signals, growth factors, and ROS triggers up-regulation of NF-κB and TNF-α, which interfere with apoptosis [8,73]. Cycles of regeneration lead to dysregulation of the cell cycle and accumulation of chromosomal instability resulting in irreversible genetic and epigenetic changes that precede neoplastic transformation [8]. Progression of malignant clones occurs through local expansion, intrahepatic spread through the portal tract, and extrahepatic metastasis. Progression is driven partly by constitutive activation of growth factors [76] as well as neo-angiogenesis, leading to an unusually high microvessel density [77].

### 2.1. Fibrogenesis

**Figure 2:**
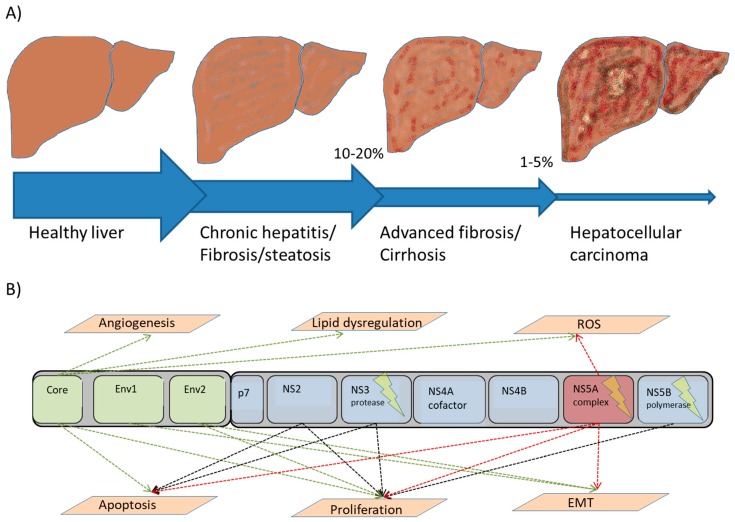
Hepatitis C virus-induced hepatocarcinogenesis. (**A**) HCV-induced hepatocarcinogenesis proceeds slowly over a span of 20–40 years accompanied by progressive fibrogenesis and eventually cirrhosis. (**B**) Both structural (Core, E1, E2) and non-structural proteins (NS2, NS3, NS5A, NS5B) play direct or indirect roles in hepatocarcinogenesis through oxidative stress, proliferation, apoptosis, chronic inflammation, dysregulated lipid metabolism, and angiogenesis. DAA targets are indicated using lightning symbols. ROS: reactive oxygen species; EMT: epithelial/mesenchymal transition.

Development of HCC is tightly coupled to fibrogenesis. Hepatic fibrogenesis serves as a wound-healing mechanism in response to injury that, in spite of the regenerative capacity of the liver, gradually results in scarring and ultimately cirrhosis (Figure 2) [78]. Although the degree of liver fibrosis typically improves following SVR, the risk of further fibrosis progression leading to cirrhosis and HCC remains [79]. In response to hepatocyte cell death, the presence of ROS, and secretion of growth factors, cytokines, chemokines, and adipokines by various cells, including hepatocyes, Kupffer cells, cholangiocytes, sinusoidal epithelial cells, and infiltrating immune cells, hepatic stellate cells become activated and begin to drive the process of fibrogenesis [80,81,82,83]. Activated hepatic stellate cells differentiate into myofibroblasts through epithelial to mesenchymal transdifferentation (EMT), in which epithelial cells acquire characteristics associated with mesenchymal cells, resulting in disruption of cell junctions and increased cell mobility [84,85]. HCV proteins directly influence TGF-β expression, a key of regulator of EMT, and both HCV core protein and NS5A promote EMT-mediated fibrogenesis via CTCF and Twist2, respectively [86,87,88].

### 2.2. Genetic Instability and Mutagenesis

Transformation of normal hepatocytes to cancer cells during hepatocarcinogenesis is accompanied by characteristic patterns of somatic mutations in, e.g., *P53*, *PIK3CA*, and *CTNNB1*, that promote genetic instability and uncontrolled proliferation [89,90,91]. Tumor cells have been found to differ from adjacent non-tumor tissue on the order of 50,000 single nucleotide variants that might affect any of nearly 1000 oncogenes and tumor suppressors [92,93]. After multiple rounds of regeneration, telomere shortening triggers cellular senescence via *ATM*, *TP53*, and *CDKN1A* [23,94,95], resulting in reduced hepatocyte proliferation. The HCV core protein is able to subvert this process through methylation-induced down-regulation of *CDKN2A* [96], resulting in increased telomerase activity. Somatic mutations in the *TERT* promoter frequently occur early during hepatocarcinogenesis [97]. HCV indirectly promotes mutagenesis by inducing oxidative stress and production of ROS, which results in damage to chromosomal and mitochondrial DNA and introduction of mutations during DNA repair [71,98], and by inducing ER stress, which facilitates accumulation of mutagenic factors [99]. Normally cell cycle checkpoint mechanisms detect and respond to severe DNA damage by triggering apoptosis, and apoptosis levels indeed remain high during fibrinogenesis but begin to flag as HCC development proceeds [100]. As in many types of cancer, the critical tumor suppressor gene *p53*, which integrates signals affecting senescence, cell-cycle arrest, and apoptosis, is frequently mutated. In addition, HCV core, NS3, and NS5A proteins have been shown to directly disrupt the p53 pathway, inhibit p53-mediated apoptosis, and promote relocation of p53 from the nucleus to the cytoplasm [101,102,103,104].

### 2.3. Apoptosis and Cell Cycle Dysregulation

HCV also blocks TNF-α-mediated apoptosis; the core protein induces *c-FLIP*, which inhibits caspase-8 activation [105], while NS5A blocks caspase-3 activation, inhibits cleavage of PARP1, and activates calpain [106,107,108]. Similarly, the core protein favors increasing polyploidy by interfering with the Rb-mediated mitotic spindle checkpoint [109]. Somatic mutations, structural alterations, and deletions affecting oncogenes and tumor suppressor genes such as *ARID2* accumulate and drive further carcinogenesis [110]. β-catenin levels are often elevated in patients with HCV-related HCC. Binding of Wnt ligands to the Frizzled receptor activates the Wnt pathway, thereby inhibiting the degradation of β-catenin, which translocates to the nucleus and up-regulates pro-tumor genes involved in cell survival and proliferation such as *c-myc* and cyclin D [111]. Mutations in the *CTNNB1* gene that encodes β-catenin are mutated in 30% of HCC cases [112] and act by stabilizing β-catenin and inhibiting its degradation [112]. Expression levels of many genes and microRNAs, including *CDKN2A*, *GSTP1*, *RUNX3*, *APC*, *SOCS*-1, *RASSF1A*, *EZH2*, and *PP2Ac* are also dysregulated through methylation or other epigenetic mechanisms [113].

### 2.4. Viral Factors

HCV proteins affect HCC development either directly or indirectly via immune-mediated inflammation. In addition to promoting inflammation and fibrogenesis via ROS [114], HCV proteins have been shown to demonstrate direct carcinogenic effects [115] and induce HCC through direct interaction with apoptosis, DNA repair, EMT, cell proliferation, metabolism, and oxidative/ER stress pathways (Figure 2B) [84]. HCV promotes cell division by disrupting RAF/MAPK/ERK-regulated pathways, activating cyclin/Cdk complexes that stimulate the transition from G1 to S phases [116]. The viral NS5B protein also stimulates cell cycle progression by binding to and promoting the degradation of Rb, which in turn up-regulates E2F-responsive genes [117,118]. The HCV core protein interacts with the mitochondrial membrane, interfering with antioxidant enzymes and the electron transport chain, and triggering the release of ROS [119,120]. As a result, liver ROS levels are elevated in HCV patients [121,122]. Similarly, HCV NS5A also promotes ROS release via calcium release from the ER [123]. HCV proteins hijack the lipid synthesis pathway by promoting lipid synthesis while reducing secretion of very low density lipoproteins, resulting in steatosis [124,125]. HCV also interferes with insulin signaling by degrading IRS-1 and IRS-2 [126]. The HCV core and NS5A proteins target the TGF-β pathway, and serum TGF-β levels have been found to be elevated in HCV patients [127]. While TGF-β has antiproliferative and proapoptotic properties, it also plays a role in EMT, and HCV-mediated inflammation is thought to shift the activity of TGF-β to a pro-fibrogenic role by blocking heterodimerization of Smad2/Smad3 and preventing nuclear translocation of Smad2, which in turn down-regulates *CDKN1A* expression [128,129]. The E1/E2 proteins play several roles in immune evasion, including interfering with interferon signaling by inhibiting PKR [130] and interfering with activation of NK cells and T cells [131].

HCV infection also brings about epigenetic changes that pass from infected hepatocytes to their daughter cells, resulting in profound effects on the development of HCC even after successful eradication of the virus. While cancer is a genetic disease driven in part by accumulation of somatic mutations, epigenetic changes are also capable of causing heritable changes in gene expression and activity by altering DNA accessibility. Addition of methyl groups to promoters by DNA methyltransferases acts to repress gene expression without altering the DNA sequence, although spontaneous deamination of 5-methylcytosine causes a mismatch and may be a source of mutations if repaired incorrectly. Methylation of hotspots on chromosomes 3 and 16 represent early events in hepatocarcinogenesis and lead to loss of heterozygosity and suppression of the tumor suppressor genes *RASSF1A*, *BLU*, and *FHI* [132]. Genome-wide methylation studies have shown that *HIST1H2AJ* and *SPDYA* are frequently methylated while *HRNBP3* is frequently hypomethylated in HCC [133]. Hypermethylation of other important cancer-related genes, including *p16*, *APC*, *p73*, *p14*, *p15*, *O^6^MGMT*, *IGF2*, *SOCS*-1, *Gadd45*, and *STAT1* have also been observed in HCV infection and were shown to be useful in prediction of HCC occurrence and survival [134,135,136,137,138]. For example, Zekri et al. proposed a panel of four genes (*APC*, *p73*, *p14*, and *O^6^MGMT*) associated with high rates of promoter hypermethylation as a biomarker for early detection of HCV-related HCC [136]. MicroRNAs contribute to hepatocarcinogenesis in part by directly targeting DNA methyltransferases, and hypermethylation of promoter CpG islands plays an important role in silencing of tumor suppressor genes such as *INK4a* and *INK4b* [132]. Methylation-related epigenetic changes in HCC are most strongly associated with the Ras/Raf/MAPK and mTOR pathways and in particular with G-protein receptor signaling [133]. Epigenetic regulation also involves modification of histone proteins. Addition of methyl groups to histone tails by histone methyltransferases suppresses gene expression by restricting DNA accessibility, whereas histone acetyltransferases increase DNA accessibility by reducing the positive charge, thereby relaxing histone affinity for negatively charged DNA. 18.2% of HCV-associated HCC cases in the United States and Europe have been found to carry inactivating mutations in the chromatin remodeling protein ARID2 [110]. Inactivation of ARID2 promotes histone deacetylation and enhances formation of E2F1 and RNA Pol II complexes, promoting *cyclin D1* and *cyclin E1* transcription. NS5A binds to and sequesters nucleosome assembly protein 1-like 1 (NAP1L1), another protein involved in chromatin remodeling, thereby impairing RIG-I and Toll-like receptor 3 (TLR3) responsiveness and possibly contributing to chronic infection and the development of HCC [139].

While each of the HCV proteins collectively helps to establish an environment predisposed to HCC development, the core and NS5A proteins each play several specific roles. In addition to regulating HCV RNA replication and translation, the core protein inhibits NF-kB-mediated host immune responses [140], activates the lipogenic pathway [141], induces ER stress via ROS [142], activates the MAPK and AP1 pathways [143], promotes cell cycle transition by up-regulating levels of cycle E/Cdk2 [144], and either directly or indirectly inhibits *TP53*, *TP73*, *RB1*, and *CDKN1A* [101,145,146]. The core protein undergoes frequent mutations and is a major risk factor for HCC progression.

DAA agents targeting the viral protein NS5A have picomolar efficacy but are nonetheless highly susceptible to resistance associated variants, which may already be present in some treatment-naive patients or emerge during the course of treatment [147]. Despite having no known enzymatic activity, the NS5A protein is thought to function as a transcriptional activator that plays key roles in HCV replication as well as in modulating the host cell innate immune response [148,149] by, e.g., activating STAT3 and NF-κB [123] and inhibiting TNF-α-driven apoptosis [106]. The viral protein causes chromosomal instability via aberrant mitosis by down-regulating the mitotic spindle regulator *ASPM* [150] and inducing oxidative stress [123]. NS5A directly up-regulates β-catenin [151,152] and activates the WNT signaling pathway by down-regulating *PTEN*, which results in activation of the PI3K/Akt survival pathway [153] and evasion of apoptosis [154]. NS5A also promotes cell proliferation by blocking TGF-β signaling via interaction with TGFBR1 and by inhibiting nuclear translocation of SMAD [129]. The protein binds to and promotes degradation of retinoblastoma, which in turn relieves repression of E2F, promoting cell cycle progression and entry into the S-phase [118].

The presence of specific viral polymorphisms in HCV core, NS3, and NS5A proteins are associated with an elevated risk of HCC [155,156], and risk of HCC is almost four-fold higher in genotype 1 than genotype 2 [157], with highest incidence associated with genotype 1b [158]. Quasispecies composition varies even within the livers of patients with HCC, suggesting that selection of HCV variants plays a role in development of HCC [159].

### 2.5. Immune-Mediated Mechanisms

The immune response plays a critical role in determining whether chronic HCV infection is established following acute infection, and the state of the immune system following SVR likely determines the risk of HCC. Remarkably, HCV produces 10^12^ virions per day while remaining noncytolytic and inducing minimal immunopathology [160]. To accomplish this feat, HCV modulates both the innate and adaptive immune responses through a range of mechanisms that disrupt homeostasis and establish an environment tolerant of viral replication but with impaired ability to detect and destroy neoplastic cells. HCV NS3 directly interferes with type I interferon production by cleaving MAVS [161]. The presence of HCV induces aberrant expression of pro-inflammatory cytokines such as TNF-α, IL-1, IL-23, IL-6, and LT-α and LT-β that help to establish and maintain a state of chronic inflammation and which have been implicated in HCC progression [75,162,163], although, interestingly, elevated serum IL-6 levels are associated with an increased risk of HCC in females but not in males due to an estrogen-dependent effect of pro-inflammatory cytokines on HCC risk [75]. The lymphotoxins LT-α and LT-β, in turn, activate NF-κB, which further amplifies cytokine and chemokine production, triggering hepatocarcinogenesis through cycles of cell death, tissue remodeling, and infiltration of lymphocytes [163]. While T and B cells are recruited to the liver, they not only fail to clear the virus but also accumulate in the liver, promoting further inflammation [164]. In fact, the presence of elevated CD8+ T-cells in the liver is associated with lower counts of NK and NKT cells, which play an important role in anti-cancer immune surveillance, and is associated with increased risk of HCC recurrence after surgical resection [164]. HCV also blunts the effectiveness of cytotoxic CD8+ T and NK cells by driving CD4+ T-cell differentiation into Th2, Th17, and Treg cells [165,166,167,168,169]. Following the rapid post-SVR decrease in viral load, a number of immune-related changes occur, including restoration of HCV-specific CD8+ T cell function, re-differentiation of memory T cells, and deactivation of lymphocytes [49]. In some cases, this rapid change in antiviral state has been linked to reactivation of other co-infecting viruses, suggesting a diminished capacity for immune surveillance and control that might fail to defend against neoplastic transformation [49].

### 2.6. Host Factors

HCC risk is 2–4 times higher in men than in women and varies with respect to obesity, family history, diabetes, alcoholism, and coinfection with HIV or HBV [40,170]. In Japan, variants in the *DEPDC5* [171] and *MICA* [172] or *HCP5* [173] loci are associated with increased risks of HCV-related HCC, although the mechanism is unclear. In a recent genome-wide association study, Matsuura et al. found a strong association between a variant in an intron of the *TLL1* gene and risk of HCC in HCV patients who achieved SVR. The authors proposed that the variant might affect splicing of the *TLL1* gene, resulting in expression of a more active form that is associated with accelerated fibrogenesis [174]. Host genetic factors in several cytokines and cytokine receptors, including polymorphisms in *TNF-α*, *IL-10*, *IL-23R*, *TLR4*, and *VEGF*, also contribute to HCC risk [175,176]. Patients with haplotypes associated with reduced production of TNF-α and IL-10 are at higher risk of HCC [177], whereas patients with a rare variant in *IL-23R* have lower risk of HCC [178]. Based on a retrospective study of patients treated for HCV infection in Taiwan, a polymorphism in *IFNL3* (formerly known as *IL28B*) was found to be an independent risk factor for development of HCC following treatment. *IFNL3* rs12979860 C/T and T/T genotypes are associated with increased risk of HCC, especially in patients who did not achieve SVR [179]. Another study showed that HCV patients with the T/T genotype have higher rates of advanced inflammation and fibrosis [180,181], increasing the risk of cirrhosis or HCC, especially in patients who did not achieve SVR. A polymorphism in the untranslated region of *IFNL3* affects transcript stability by influencing the binding affinity of microRNAs induced by HCV infection, resulting in higher risk of HCC [182].

## 3. Experimental Systems

The high rate of HCC mortality is partly attributable to lack of biomarkers and incomplete knowledge of the molecular pathways deregulated early during hepatocarcinogenesis and how to target them, resulting in late detection and few effective or durable treatment options [183,184]. A number of biomarkers have been investigated, including alpha-fetoprotein (AFP), AFP-L3, des-γ-carboxyprothrombin (DCP), glypican-3, cytokeratin 19, Golgi protein 73 (GP73), midkine (MDK), osteopontin, squamous cell carcinoma antigen (SCCA), and annexin A2; however, clinical studies often provide conflicting results, suggesting that much work remains in order to identify biomarkers with sufficient sensitivity and specificity [185]. More complex multivariable models that better reflect underlying metabolic changes offer a promising approach. For example, Hoshida et al developed a 186-gene signature from needle biopsy samples in patients with HCV-related Child-Pugh class A cirrhosis to predict prognosis and prioritize surveillance efforts [186]. Similarly, Huang et al. reported a panel of 5 serum microRNAs capable of classifying cirrhotic patients into high and low-risk groups [187]. Such approaches might also be used to predict response to specific therapies. However, HCC tumors demonstrate extensive sequence heterogeneity, limiting the effectiveness of drugs that target specific mutations, and it remains unclear whether the various etiologies of HCC ultimately converge into a single HCC-specific signature. In vitro and in vivo models are needed to answer these questions, although each model suffers from specific limitations. However, evidence from a number of mouse models suggests that HCC tumors from animal models are histologically and molecularly similar to human tumors and may be characterized by specific gene expression profiles distinct from other cancer types and without regard to etiology [184]. Nonetheless, etiology-specific differences have also been found, which might limit the degree to which results may be assumed to apply to etiologies that are more difficult to model experimentally.

### 3.1. Cell Models

Hepatoma cell lines already play a critical role in studies to unravel the molecular mechanisms of HCV due to the difficulty of establishing infection in primary hepatocytes, but adaption to cell culture necessarily limits the generality of the conclusions that can be drawn. Saeed et al. identified SEC14L2 as a factor required to support pan-genotypic HCV replication in Huh-7.5 cells without compensatory cell culture adaptive mutations [188], although it was later found that only 7 out of 13 SEC14L2 variants enabled HCV replication, and SEC14L2 only enhanced the transcription of genotypes 1 and 3 but not genotypes 2, 4, and 5. Despite these limitations, SEC14L2-expressing Huh7.5 cell lines provide a model to study the mechanism by which host genes facilitate HCV replication and a platform for drug screening.

In order to examine the effect of tumor heterogeneity on drug efficacy, Hirschfield et al. compared the transcriptomes of multiple commonly used hepatoma cell lines and found that gene expression patterns corresponded to HCC subtypes S1 and S2 but not the less aggressive S3 subtype, suggesting that these cell lines can serve as tractable models for drug testing in different HCC subtypes [189].

### 3.2. Animal Models

Animal models are invaluable in uncovering the steps involved in HCC development and identifying biomarkers that might allow earlier detection of HCC [184,190,191]. Chimpanzees were initially used to study HCV infection due to their close relatedness to humans and the lack of a suitable small animal model. In spite of the limitations due to availability, cost and, ethical considerations, chimpanzees played a key role in the identification of host-virus interactions and screening of HCV vaccine candidates [192]. However, in a longitudinal study in which chimpanzees were inoculated with plasma from a patient with chronic HCV, only one occurrence of HCC was observed after 7 years, making it a poor model for studying HCV-related hepatocarcinogenesis [193]. Mouse models are widely used to study the complete life cycle of HCV and HBV and provide a system for evaluating the effect of antiviral therapies [194,195], and a number of transgenic mouse models have been generated to model HCC. Some models induce HCC chemically using aflatoxin or other carcinogens, while other models have been engineered to over-express factors such as TNF-α, c-Myc, HCV core protein, HBxAg, or HBsAg [196,197,198,199,200]. Teoh et al. combined these approaches by exposing transgenic HBsAg carrier mice to aflatoxin and observed a synergistic effect [184]. While many of these models closely approximate human HCC and have contributed to understanding of hepatocarcinogenesis, they do not fully capture each of the molecular and cellular changes known to occur during transformation.

## 4. Conclusions and Future Perspective

Despite optimism surrounding the remarkable success of DAA, many patients who have been successfully cured of HCV infection will go on to face HCC at some point during the following decade. Fortunately early concerns that DAA therapy left patients at higher risk than patients treated with interferon therapy have largely been addressed, but cost and availability of DAA therapies prevent many high-risk individuals in underserved areas from receiving this state of the art treatment. HCV-related HCC development is a complex process occurring over the span of decades and involves the slow remodeling of the liver into a microenvironment that is optimized for HCV replication but is also predisposed to cellular transformation and carcinogenesis. The specific mechanisms of post-SVR HCC remain to be determined but likely involve the nature of the immune response and epigenetic state in the liver following successful treatment. Improvements in in vitro and in vivo models are anticipated to lead to development of more accurate biomarkers for early detection and more effective therapies designed to target the HCC-specific and etiology-specific pathways alterations implicated in progression of this deadly and ascendant form of cancer.

## Figures and Tables

**Table 1:** Studies reporting incidence of hepatocellular carcinoma following post-sustained virological response in interferon-treated chronic hepatitis C patients (adapted from Baumert et al., 2017 [49]). Prospective studies are indicated using bold text.

Reference	Country	*N*	Follow-Up (Months)	Males (%)	Age	Cirrhosis (%)	Post-SVR HCC (%) *
Akuta (2011) [50]	Japan	1273	1.1	61.5	53	8.6	3.2
Chang (2012) [51]	Taiwan	1271	3.4	75.9	55	27.9	1.2
Huang (2014) [52]	Taiwan	642	4.4	54.3	51	13.4	5.8
**Oze (2014) [53]**	**Japan**	**1425**	**3.3**	**51.0**	**55**	**11.6**	**2.6**
Saito (2014) [54]	Japan	14	3.9	92.9	72	85.7	18.0
Yamashita (2014) [55]	Japan	562	4.8	55.3	57	23.0	3.1
Huang (2015) [56]	Taiwan	56	4.4	64.3	62	37.5	43.2
Toyoda (2015) [57]	Japan	522	7.2	55.9	51	5.5	1.2
El-Serag (2016) [40]	USA	10,738	2.8	95.3	53	14.4	0.3
Kobayashi (2016) [58]	Japan	528	7.3	58.4	54	14.8	2.2
Kunimoto (2016) [59]	Japan	40	5.1	87.5	65	35.0	23.0
Minami (2016) [60]	Japan	38	-	71.0	66	0	52.9
Nagaoki (2016) [61]	Japan	1094	4.2	53.5	60	1.9	4.0
Tada (2016) [62]	Japan	587	14.0	55.2	50	-	4.4
Tada (2016) [62]	Japan	170	14.2	62.4	53	-	7.1
van der Meer (2016) [63]	EU, Canada	1000	5.7	68.0	53	85.0	7.6
Wang (2016) [64]	Taiwan	376	7.6	49.2	54	33.8	1.4
Nagata (2017) [43]	Japan	1145	6.8	54.0	59	-	2.6
Kobayashi (2017) [58]	Japan	77	4.0	44.2	63		3.0
Petta (2017) [65]	Italy	57	2.8	72.0	62	0	15.0
Motoyama (2018) [45]	Japan	11	8.1	81.0	55	36.3	-

* Overall reported rates of HCC following SVR during the follow-up period.

**Table 2:** Studies reporting incidence of hepatocellular carcinoma following post-sustained virological response in chronic hepatitis C patients treated using direct-acting antiviral therapy (adapted from Baumert et al., 2017 [49]). Prospective studies are indicated using bold text.

Reference	Country	*N*	Follow-Up (Months)	Males (%)	Age	Cirrhosis (%)	Post-SVR HCC (%) *
**ANRS (2016) [42]**	**France**	**189**	**2.2**	**78.0**	**62**	**80.0**	**0.7**
**ANRS (2016) [42]**	**France**	**13**	**1.8**	**85.0**	**61**	**100.0**	**1.1**
**ANRS (2016) [42]**	**France**	**314**	**-**	**82.0**	**61**	**15.6**	**2.2**
Cardoso (2016) [66]	Portugal	54	1.0	76.0	59	-	7.4
**Cheung (2016) [32]**	**UK**	**317**	**1.3**	**-**	**54**	**80.1**	**5.4**
Conti (2016) [31]	Italy	344	0.5	60.1	63	11.3	3.2
Conti (2016) [31]	Italy	59	0.5	67.8	72	16.9	28.8
Kobayashi (2016) [58]	Japan	77	4.0	44.2	63	29.9	3.0
Kozbial (2016) [34]	Austria	19	-	73.7	-	73.7	50.0
Minami (2016) [60]	Japan	27	-	67.0	71	0	29.8
Petta (2016) [65]	Italy	58	1.5	69.0	66	4.0	26.3
Reig (2016) [30]	Spain	58	0.5	69.0	66	8.6	27.6
Calleja (2017) [67]	Spain	1567		53.7	60	46.7	0.9
Nagata (2017) [43]	Japan	752	1.8	45.0	69	-	3.3
Kanwal (2017) [46]	US	22,500	2.0	96.7	62	68.7	0.9
Kobayashi (2017) [58]	Japan	528	7.3	58.4	54		2.2
**Mettke (2017) [68]**	**Germany**	**158**	**1.2**	**55.0**	**59**	**100.0**	**2.9**
Petta (2017) [65]	Italy	58	1.5	69.0	66	-	10.8
**Calvaruso (2018) [69]**	**Italy**	**2249**	**1.1**	**56.9**	**65**	**-**	**3.0**

* Overall reported rates of HCC following SVR during the follow-up period.
